# Simultaneous Assay of CA 72-4, CA 19-9, CEA and CA 125 in Biological Samples Using Needle Three-Dimensional Stochastic Microsensors

**DOI:** 10.3390/s23198046

**Published:** 2023-09-23

**Authors:** Alexandru-Adrian Bratei, Raluca-Ioana Stefan-van Staden, Ruxandra-Maria Ilie-Mihai, Damaris-Cristina Gheorghe

**Affiliations:** 1 Laboratory of Electrochemistry and PATLAB, National Institute of Research for Electrochemistry and Condensed Matter, 202 Splaiul Independentei Str., 060021 Bucharest, Romania; brateialexandru@yahoo.com (A.-A.B.); i.ruxandra04@yahoo.com (R.-M.I.-M.); gheorghe.damaris16@gmail.com (D.-C.G.); 2 Faculty of Chemical Engineering and Biotechnologies, National University of Science & Technology Politehnica Bucharest, 060021 Bucharest, Romania; 3 Department of Pathology, Emergency University Hospital, 050098 Bucharest, Romania; 4 Department of Pathology, George Emil Palade University of Medicine, Pharmacy, Sciences and Technology, 540139 Targu Mures, Romania

**Keywords:** gastric cancer, stochastic sensor, CA 72-4, CA 19-9, CEA, CA 125

## Abstract

Two-needle 3D stochastic microsensors based on boron- and nitrogen-decorated gra-phenes, modified with N-(2-mercapto-1H-benzo[d]imidazole-5-yl), were designed and used for the molecular recognition and quantification of CA 72-4, CA 19-9, CEA and CA 125 biomarkers in biological samples such as whole blood, urine, saliva and tumoral tissue. The NBGr-2 sensor yielded lower limits of determination. For CEA, the LOD was 4.10 × 10^−15^ s^−1^ g^−1^ mL, while for CA72-4, the LOD was 4.00 × 10^−11^ s^−1^ U^−1^ mL. When the NBGr-1 sensor was employed, the best results were obtained for CA12-5 and CA19-9, with values of LODs of 8.37 × 10^−14^ s^−1^ U^−1^ mL and 2.09 × 10^−13^ s^−1^ U^−1^ mL, respectively. High sensitivities were obtained when both sensors were employed. Broad linear concentration ranges favored their determination from very low to higher concentrations in biological samples, ranging from 8.37 × 10^−14^ to 8.37 × 10^3^ s^−1^ U^−1^ mL for CA12-5 when using the NBGr-1 sensor, and from 4.10 × 10^−15^ to 2.00 × 10^−7^ s^−1^ g^−1^ mL for CEA when using the NBGr-2 sensor. Student’s *t*-test showed that there was no significant difference between the results obtained utilizing the two microsensors for the screening tests, at a 99% confidence level, with the results obtained being lower than the tabulated values.

## 1. Introduction

Recently, the interest in using biomarkers to predict the presence of cancer at an early stage and to follow real-time cancer dynamics has been increasing as the currently used methods are mostly invasive and expensive [1,2]. Gastric cancer is one of the most aggressive malignancies, being the fifth most common cancer and third in the list of death-causing cancers worldwide [3]. There are nearly one million new cases of gastric cancer reported each year [4]. Four of the most-used indicators for gastric cancer are CA 72-4, CA 19-9, CA 125 and CEA [5,6,7] and they are the most-used markers for combined diagnostic models [8]. CA 72-4 is a tumor-associated epithelial mucin that is expressed in adenocarcinomas such as gastric, colonic and breast, but is lower in normal tissues [9,10,11]. In digestive system tumors, CA 72-4 is associated with high sensitivity and specificity [12,13]. CA 19-9, carbohydrate antigen 19-9 or sialyl Lewis antigen, is a tumor biomarker largely used for the screening and identification of pancreatobiliary cancer, but also for digestive tract carcinomas, especially for gastric ones [14,15,16,17]. CA 125 is a glycoprotein present in the epithelium lining body cavities, and is linked with high sensitivity and specificity in the diagnosis of several digestive tract carcinomas [18,19]. CEA is an intracellular glycoprotein that is produced by epithelial tumor cells, being an adhesion molecule, and helps with angiogenesis. Its serum level is increased in colorectal, gastric and other adenocarcinomas [20,21,22,23], but also in non-malignant conditions, such as chronic inflammatory bowel disease, smoking, alcoholism and livers disease [24,25,26].

In recent years, a large series of studies have been conducted for the detection and quantification of the four biomarkers individually or in combination using the electrochemiluminescence immunoassay (ECLIA), enzyme-linked immunosorbent assay (ELISA), chemiluminescence immunoassay (CLIA), quantitative reverse transcription polymerase chain reaction (qRT-PCR) and Luminex xMAP [27,28,29,30,31,32,33,34,35,36,37,38,39]. The methods mentioned earlier are characterized by their labor-intensive nature, as they require the individual determination of each biomarker. Additionally, it is worth noting that the cost per analysis is relatively expensive.

Therefore, this article proposes a screening method for whole blood, saliva, urine and gastric tumor tissues that is able to simultaneously recognize and quantify the following four biomarkers: CA 72-4, CA 19-9, CA 125 and CEA. The proposed tools are stochastic sensors. The advantages of using stochastic sensors are as follows: they can perform the molecular recognition as well as the quantitative analysis of more biomarkers in a single run; the response of the stochastic sensors is not influenced by the complexity of the matrix from which the biomarkers are analyzed; the method is cost-effective; and the sensors can usually be used for more than 100 analyses of a panel of biomarkers from biological samples. The novelty of the method is represented by the design of the 3D needle stochastic microsensor (based on graphenes decorated with B and N specially synthesized for this purpose, modified with an oleamide that is able to provide the necessary channels for the stochastic response), and by the approach of simultaneously analyzing CA 72-4, CA 19-9, CA 125 and CEA with high reliability. The conductivity of the matrix material, specifically graphene, was enhanced by incorporating N- and B-doped graphene. Compared with the graphite material, where the van der Waals forces between graphene layers may modify the shape of the channels, the utilization of the graphene material proved to be significantly more convenient, because the shape of the channel is not influenced by physical forces like van der Waals. This is primarily attributed to the elevated stability of the signal and improved reproducibility of measurements. The selection of the stochastic microsensors—as screening tools for the simultaneous assay of CA 72-4, CA 19-9, CA 125 and CEA—is based on the following advantages of using stochastic sensors in biomedical analysis versus classical electrochemical sensors [40]: they are the only sensors able to perform reliable molecular recognition (qualitative analysis) when biomarkers are determined in complex matrices like biological fluids and tissues; they can perform multianalyte analysis from complex matrices; the signatures of the biomarkers are not influenced by the content of the matrix from where the determination is taking place, but they depend on the size of the biomarkers, capacity to unfold (if 2D or 3D structures are analyzed), velocity of getting inside the pore, and geometry and stereochemistry of the analyzed biomarkers. Quantitative analysis is considered highly reliable due to its use within a controlled environment, specifically within the confines of a channel or pore. For the proposed stochastic sensors, graphene is a very good matrix, able to provide stability to the channels and pores of any modifier. Oleamides are molecules that are able to provide the channels needed in the stochastic determinations. The design of the 3D needle type was selected to accommodate the tissue analysis. The proposed needle 3D stochastic microsensors are also cost-effective, and are able to analyze four biomarkers with a low cost in one run. ELISA—the standard method used to date in clinical laboratories—can only analyze one biomarker at a very high cost.

## 2. Materials and Methods

### 2.1. Reagents and Materials

The four biomarkers (CA 19-9, CA 72-4, CA 125 and CEA) and the phosphate buffer solution (pH = 7.50) were purchased from Sigma-Aldrich, while the paraffin oil was purchased from Fluka. The serial dilution method was used for the biomarkers’ solution preparation.

### 2.2. Synthesis of Boron- and Nitrogen-Decorated Graphenes

The graphene samples were obtained by electrochemically exfoliating the graphite rods, immersed in the appropriate electrolyte (100 mL). For the first sample (NBGr-1), the electrolyte contained 0.1 mol L^−1^ ammonium sulfate, 0.1 mol L^−1^ boric acid and 0.05 mol L^−1^ NaCl. For the second sample (NBGr-2) the electrolyte was made of 0.05 mol L^−1^ ammonium sulfate, 0.1 mol L^−1^ boric acid and 0.05 mol L^−1^ NaCl. The graphite rods were connected to the exfoliation system (home-made system) and a constant voltage of 12 V was applied for about 4 h between the anode and cathode. The black powder resulting from the anode exfoliation and deposited at the bottom of the cell was collected via decantation and thoroughly washed with double-distilled water (10 L). Next, the powder was dispersed via ultrasound for 30 min in 125 mL water and filtered on white-ribbon paper to remove the large particles. The last step was drying via lyophilization. Both NBGr-1 and NBGr-2 graphene powders were used in the construction of the stochastic microsensors for the simultaneous assay of CA 19-9, CA 72-4, CA 125 and CEA in biological samples.

### 2.3. Design of the Stochastic Microsensors

**Scheme 1:**
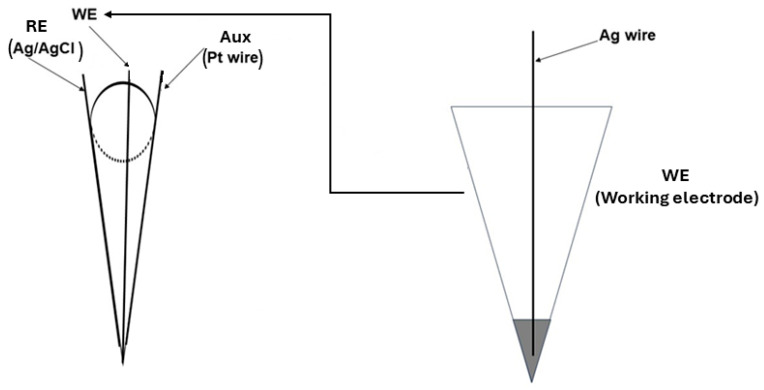
The design of the needle 3D stochastic microsensor (WE is the working electrode).

The two-needle 3D stochastic microsensors were designed as follows: each of the powders (NBGr-1 and NBGr-2) were mixed with paraffin oil until a homogeneous paste was obtained. Each of the pastes was mixed with a 10^−3^ mol L^−1^ solution of the oleamide N-(2-mercapto-1H-benzo[d]imidazole-5-yl). Three-dimensional microcones with an internal diameter of 10 μm were printed in our laboratory using a 3D Stratasys Objet 24 printer, which employs PolyJet technology for the incremental construction of three-dimensional models through a layer-by-layer process. The material employed in this study was Vero White Plus, which is a firm white opaque polymer. The support material known as FullCure 705 is an acrylic-based photopolymer with a gel-like consistency. It possesses the properties of being easily washable and non-toxic. The precision of the printer was measured to be 0.1 mm. The temperature range during operation was recorded as 18–25 °C, while the relative humidity range was measured as 30–70%. The duration required for the printing of the 3D microcones was 2 h. The glossy polymer model was fabricated and positioned in a vertical orientation on the printing table in order to minimize the utilization of the support material. The diameter of the working electrode’s surface was measured to be 10 μm. The modified pastes were placed in 3D microcones (internal diameter 10 μm) specifically designed for the needle 3D stochastic microsensors (Scheme 1).

Further, the working electrode (WE) was placed in a cone also containing the reference electrode (Ag/AgCl wire) and the auxiliary electrode (Pt wire) (Scheme 1). When not in use, the needle microsensors were kept in dry places, at room temperature.

### 2.4. Apparatus

The AUTOLAB/PGSTAT 12 Potentiostat/Galvanostat (Metrohm, Utrecht, The Netherlands) was utilized for all measurements. All measurements were performed at 125 mV vs. Ag/AgCl, and at 25 °C. The surface morphology of the pastes based on NBGr-1 and NBGr-2 were investigated using scanning electron microscopy (SEM) (Inspect S, FEI Company Netherlands). To obtain a good resolution, the following working parameters were used: LFD detector (low-vacuum mode), a high voltage (HV) of 30 kV, a value of the spot of 4, and a magnification of 3000.

### 2.5. Stochastic Method

**Figure 1:**
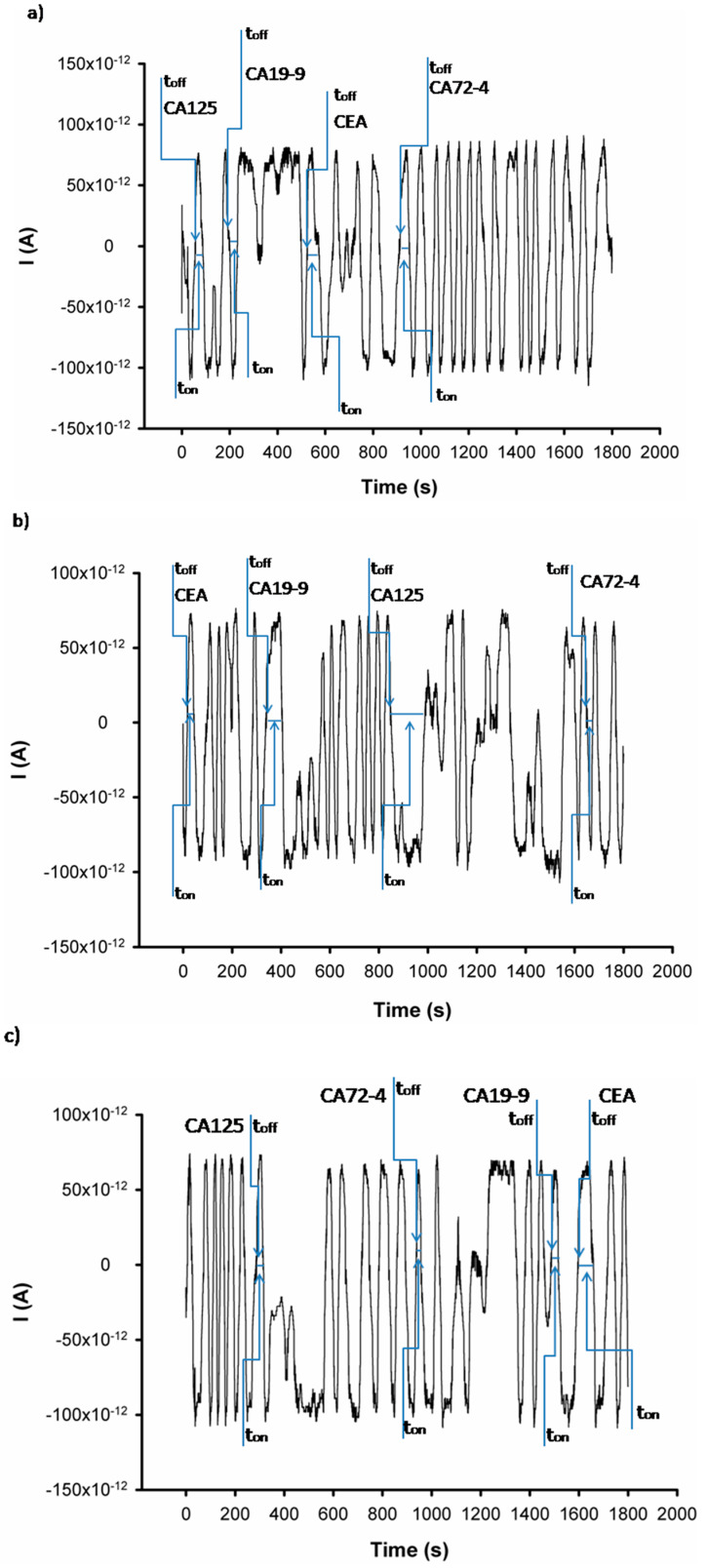

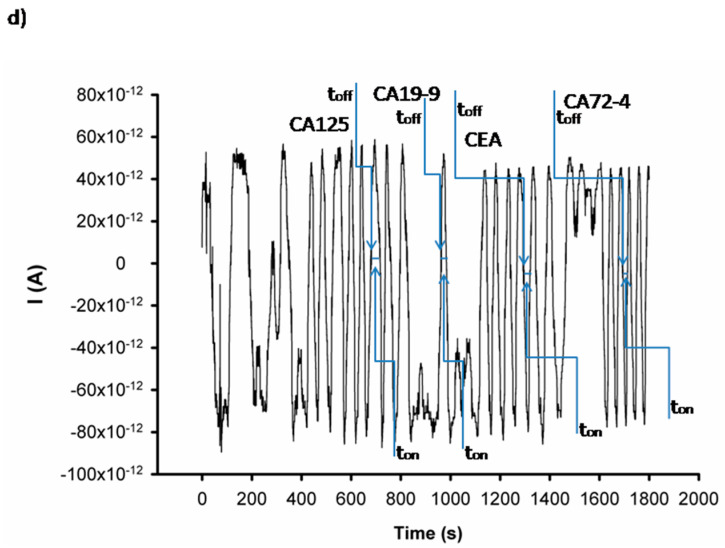
Examples of diagrams obtained when the needle 3D stochastic microsensor based on NBGr-1 was used for the screening of (**a**) saliva, (**b**) whole blood, (**c**) gastric tumor tissue and (**d**) urine.

**Figure 2:**
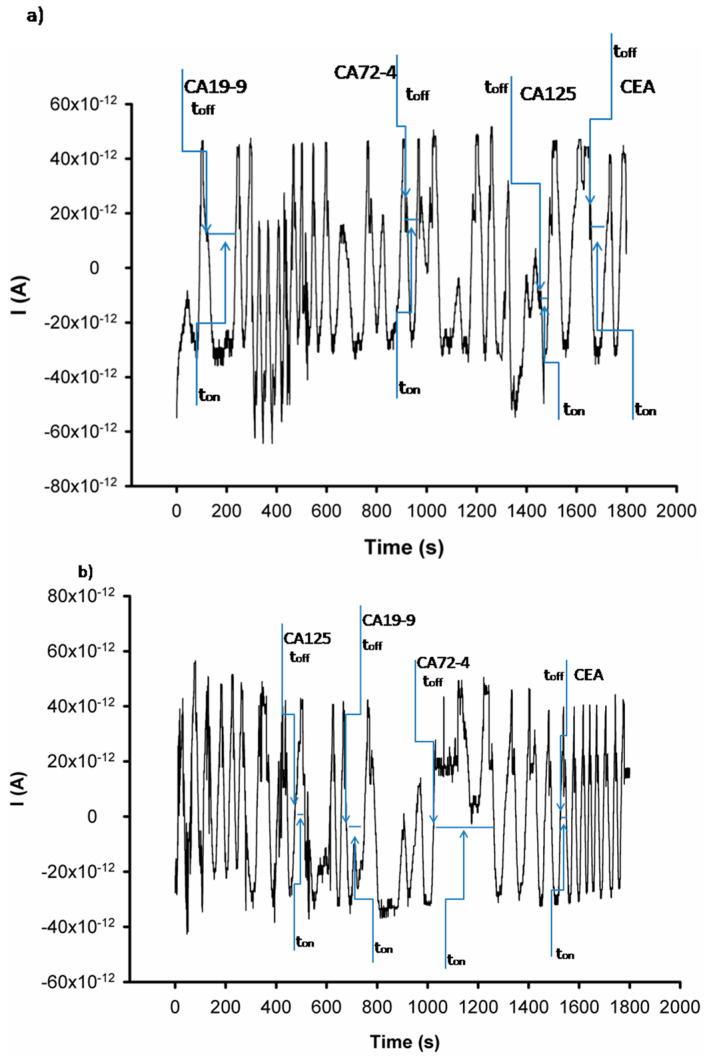

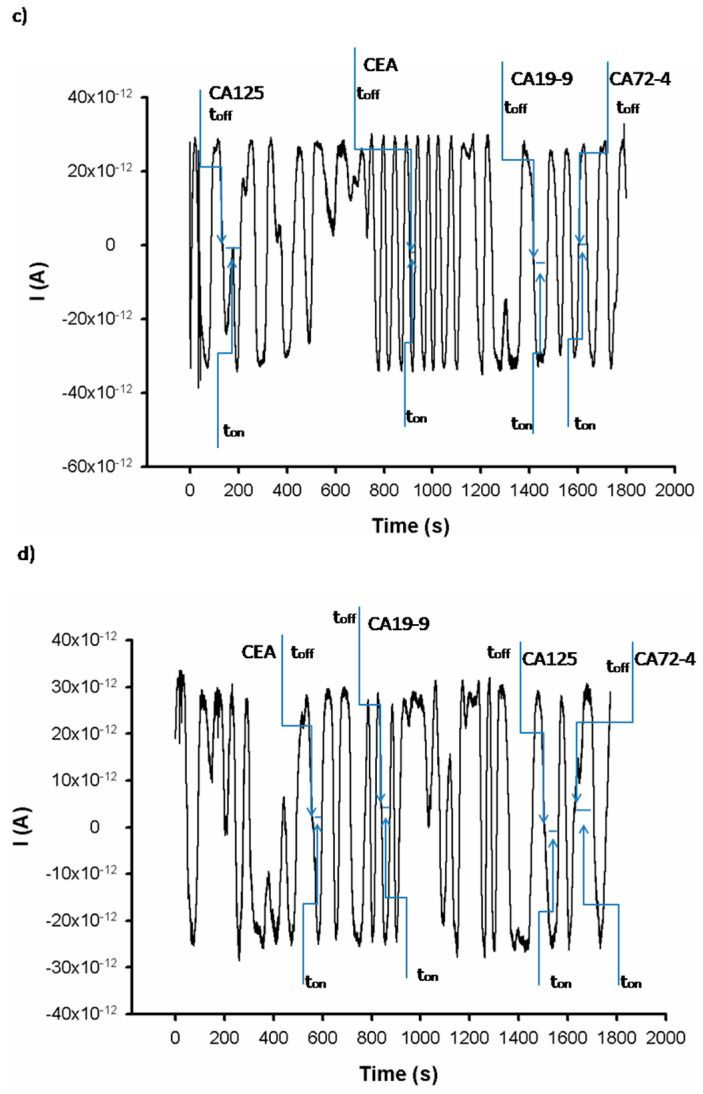
Examples of diagrams obtained when the needle 3D stochastic microsensor based on NBGr-2 was used for the screening of (**a**) saliva, (**b**) whole blood, (**c**) gastric tumor tissue and (**d**) urine.

The stochastic method involves conducting measurements of t_on_ and t_off_ at a consistent voltage (125 mV against Ag/AgCl) through the utilization of the chronoamperometric method. After conducting a thorough analysis of potentials ranging from 0 to 500 mV, a potential of 125 mV was chosen. This specific value was determined to yield readable signatures (t_off_ values) that could be consistently and accurately interpreted. The values of t_off_—also named as the signatures of the biomarkers (as based on their values, the biomarkers are recognized in the diagrams)—were used for identification of the four biomarkers (CA 72-4, CA 19-9, CEA and CA 125), and the values of t_on_ (which are read in between two signatures) were used for the determination of the concentration of each of the biomarkers using the calibration equation 1/t_on_ = a + b × C_biomarker_, where C_biomarker_ is the concentration of the biomarkers determined using the proposed 3D needle stochastic microsensors (CA 72-4, CA 19-9, CEA and CA 125), as seen in Figure 1 and Figure 2. The parameter known as “t_off_” denotes the duration required for the biomarker to enter the channel. It is commonly referred to as the biomarker’s signature and is visually indicated on diagrams using the label “t_off_”. The signature holds significant importance in qualitative analysis as it is closely associated with the molecular identification of biomarkers. Every analyte generates a distinct signature (t_off_) that is affected by factors such as its size, shape, stereogeometry, unfolding capacity and velocity when traversing the channel or pore. Consequently, it is rare for two analytes to exhibit identical signatures.

### 2.6. Samples

Four kinds of samples (saliva, whole blood, urine and tissue) were collected from patients confirmed to have gastric cancer. The patients were selected from the database of the project GRAPHSENSGASTROINTES, and their data used according to the Ethics committee approval nr. 32647/2018 awarded by the County Emergency Hospital from Targu Mures. Informed consent was obtained from all patients involved in this study. All biological samples were collected before any treatment for cancer was performed. No treatment of the sample was performed before any measurement. The samples were analyzed as soon as they were collected from the patients.

## 3. Results and Discussion

### 3.1. Morphology of the Active Surface of the Needle 3D Stochastic Microsensors

**Figure 3:**
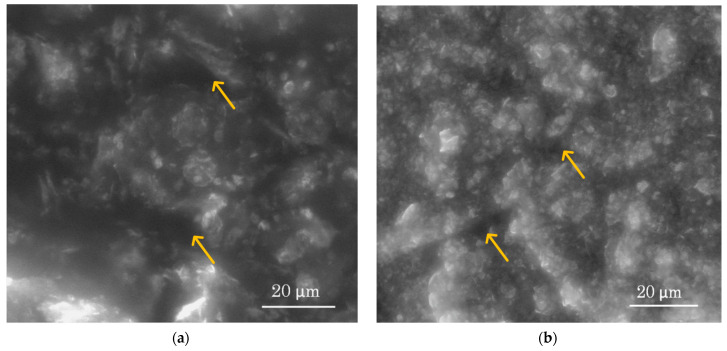
SEM images for the active side of the needle stochastic microsensor based on (**a**) NBGr-1 and (**b**) NBGr-2. Arrows are showing the presence of channels on the active surface.

Electron microscopy images for the pastes based on NBGr-1 and NBGr-2 are presented in Figure 3. The SEM images prove that there are channels on the active side of the 3D needle stochastic microsensors.

These channels are needed for stochastic sensing, and therefore, one can say that specific stochastic signals are able to be produced if the sensors are used in chronoamperometry mode. Many surface analysis studies have confirmed that the surface of the graphene is smooth and stable [41], facilitating the high stability of the modifiers’ channels. 

### 3.2. Response Characteristics of the Two Stochastic Microsensors Used for the Assay of the Four Biomarkers (CA 19-9, CA 72-4, CA125 and CEA)

The current development of stochastic sensors is a two-phase process: In the first phase, also known as the recognition phase, the biomarker enters the channel; while entering the channel, the biomarker blocks it, making the intensity of the current decrease to a value of zero) for the duration of the process of entering the channel (the time needed for the biomarker to enter the channel is called the signature of the biomarker, and it is marked on the diagrams as t_off_). The second phase is the one where the quantity of the biomarker is determined, and the t_on_ parameter is connected to the concentration according to the equation shown in the stochastic mode paragraph (see above). While the signature is very important for the qualitative analysis (being the parameter related to the molecular recognition of the biomarkers), the t_on_ parameter gives the response characteristics of the needle 3D stochastic microsensors. The response characteristics of the two-needle 3D stochastic microsensors are shown in Table 1.

Different signatures were recorded for the four biomarkers when the same microsensor was used, proving that the two microsensors can be reliably used for the simultaneous assay of the four biomarkers. High sensitivities and low limits of determination were obtained for all needle 3D stochastic microsensors. The limits of determination were determined as the lowest concentration found in the linear concentration range according to the new IUPAC recommendation (paragraph 3.36, Note 3) [42]. While the type of graphene did not significantly influence the sensitivity of the assay of the biomarkers (with the exception of CA 19-9, for which better sensitivity was recorded when NBGr-2 was used, and CEA, for which better sensitivity was recorded when NBGr-1 was used), it had more of an influence on the limits of determination of the biomarkers: lower limits of determination were obtained for CA 72-4 and CEA when the microsensor based on NBGr-2 was used, and for CA 19-9 and CA 125 when the microsensor based on NBGr-1 was used. The electrochemical reaction was induced by the applied voltage on the working electrode, and the quantity of electrons moved (referred to as electrical current) offers insights into the surface condition [43,44,45,46]. The rate of change in an electric current is directly proportional to the quantity of molecules that have undergone adsorption on the surface, hence imparting valuable sensing data. The linear concentration ranges recorded were wide, making possible the assay of the four biomarkers in healthy people, and in patients with gastric cancer from early to late stages.

Reproducibility and stability studies were performed for each of the needle 3D stochastic microsensors. Ten needle 3D stochastic microsensors from each category were designed according to the method described above, and the sensitivity values were recorded and compared for 60 days. For the reproducibility of the design, the sensitivities recorded for microsensors of the same type were compared for each biomarker; the % RSD recorded for the sensitivities of the needle 3D stochastic microsensors were as follows: for the microsensor based on NBGr-1, the values recorded were 0.03% for CA 72-4, 0.02% for CA 19-9, 0.03% for CA 125 and 0.01% for CEA, while for the microsensor based on NBGr-2, the values recorded were 0.02% for CA 72-4, 0.01% for CA 19-9, 0.04% for CA 125 and 0.01% for CEA. These values obtained for % RSD confirmed the reproducibility of the design of the two types of needle 3D stochastic microsensors.

The stability in time was determined by measuring the sensitivities of the designed sensors during 60 days. By comparing the sensitivities obtained during this period of time for each type of needle 3D stochastic microsensor, the following statements can be made: for the microsensor based on NBGr-1, the RSD values were 0.05% for CA 72-4, 0.06% for CA 19-9, 0.08% for CA125 and 0.03% for CEA, while for the microsensor based on NBGr-2, the values recorded were 0.08% for CA72-4, 0.04% for CA19-9, 0.03% for CA125 and 0.03% for CEA. These results prove the good stability of the modified pastes in time, and also of the stochastic microsensors in time.

The selectivity of stochastic microsensors is determined using the recorded values of the signatures associated with the biomarkers and other compounds present in biological samples. The presence of discernible differences between these signatures serves as evidence of the microsensors’ selectivity. The recorded toff values for various potential interferences serve as indicators of the selectivity of the two stochastic sensors under consideration. The following substances were investigated as possible causes of interference: p53, cathepsin D, cathepsin B, leucine, serine and glutamine. The signature of the four biomarkers was determined to be less than 2 s when utilizing both sensors. The other compounds present in the biological samples that were examined as potential causes of interference had characteristics distinct from the hypothesized biomarkers, hence confirming the sensors’ selectivity. When the needle 3D stochastic microsensor based on NBGr-1 was used, the following signatures were recorded: 2.4 s for p53, 2.7 s for cathepsin D, 2.9 s for cathepsin B, 3.1 s for leucine, 3.9 s for serine and 3.7 s for glutamine. When the needle 3D stochastic microsensor based on NBGr-2 was used, the following signatures were recorded: 3.8 s for p53, 3.0 s for cathepsin D, 2.8 s for cathepsin B, 3.5 s for leucine, 2.4 s for serine and 2.6 s for glutamine.

All signatures obtained for these substances were different each from each other and higher than 2.3, proving the selectivity of the proposed needle 3D stochastic microsensors when used for the assay of CA 19-9, CA 72-4, CA 125 and CEA in biological samples. Accordingly, the needle 3D stochastic microsensors can be selectively used for the assay of CA 19-9, CA 72-4, CA125 and CEA in biological samples.

In comparison to other tools and methods proposed to date (including an ultrasensitive electrochemical immune sensor proposed for the assay of CA 72-4 by Yan et al. [47]; an electrochemical sensor proposed for the simultaneous assay of CA19-9 and CA 72-4, which was based on tumor marker dual recognition via glycosyl imprinting and lectin-specific binding, proposed by Luo et al. [48]; an ultrasensitive split-type electrochemical immunosensor based on the controlled-released strategy proposed by Li et al. for the assay of CA 19-9 [49]; a photoelectrochemical immunosensor proposed by Gholamin et al. for the assay of CA 19-9 [50]; a flower-shaped chemiluminescence-based sensor for the assay of CEA [51]; a magnetic copper silicate and boronic acid conjugate AuNCs@keratin-based electrochemical/fluorescent dual sensor proposed by Jin et al. for the determination of CEA [52]; for the assay of CA 125, a hydrogel-based immunosensor proposed by Er et al. [53]; and a label-free dual immunosensor proposed by Kamac et al. [54]), the needle 3D stochastic microsensors proposed in this paper had the following advantages: The developed sensors exhibit reliable molecular recognition capabilities for CA 72-4, CA 19-9, CEA and CA 125, and they demonstrate wider working concentration ranges compared to those documented in the referenced papers. Additionally, the sensors achieve lower limits of determination and higher sensitivities, and their design, which does not involve the use of biomolecules, contributes to their enhanced stability over time. Notably, the needle 3D stochastic sensors can be stored at room temperature for a minimum of two months without compromising their functionality. Moreover, they can be utilized on a daily basis for the quantitative analysis of CA 72-4, CA 19-9, CEA and CA 125 in various biological samples, including whole blood, urine, saliva and tumor tissues. The aforementioned sensors have a higher degree of selectivity compared to biomarkers found in whole blood, saliva, urine and tumoral tissues.

### 3.3. Ultrasensitive Determination of the Four Biomarkers in All Four Biological Fluids

The wide working concentration ranges, low limits of determination, and possibility of the simultaneous determination of CA 72-4, CA 19-9, CEA and CA 125 made possible the utilization of needle 3D stochastic microsensors for screening tests of whole blood, saliva, urine and tumoral tissues.

**Figure 4:**
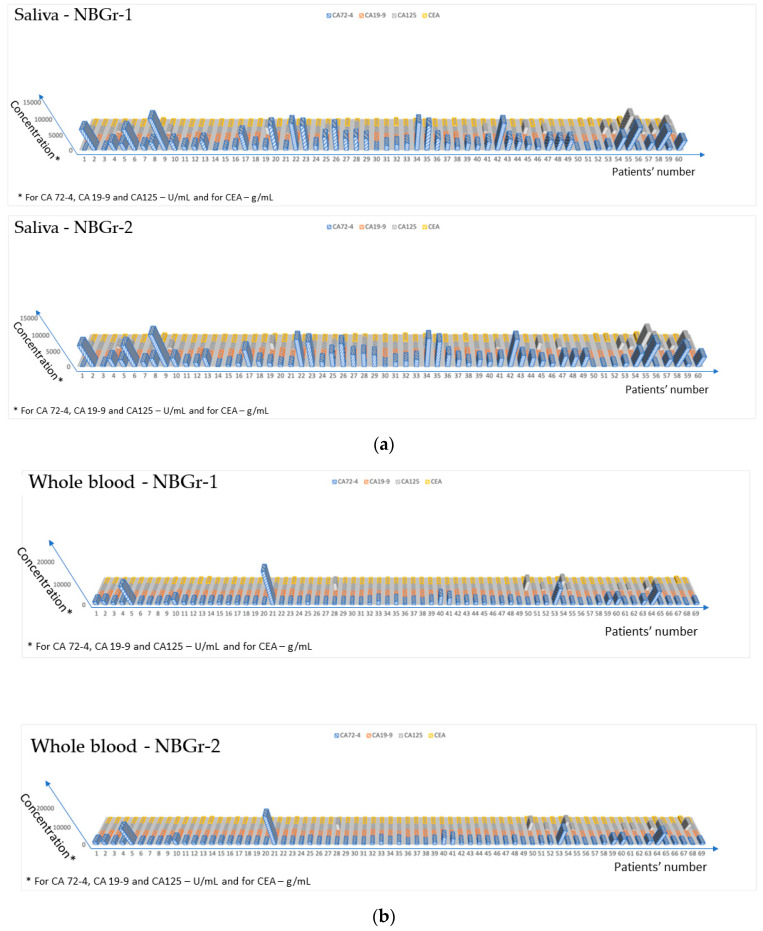

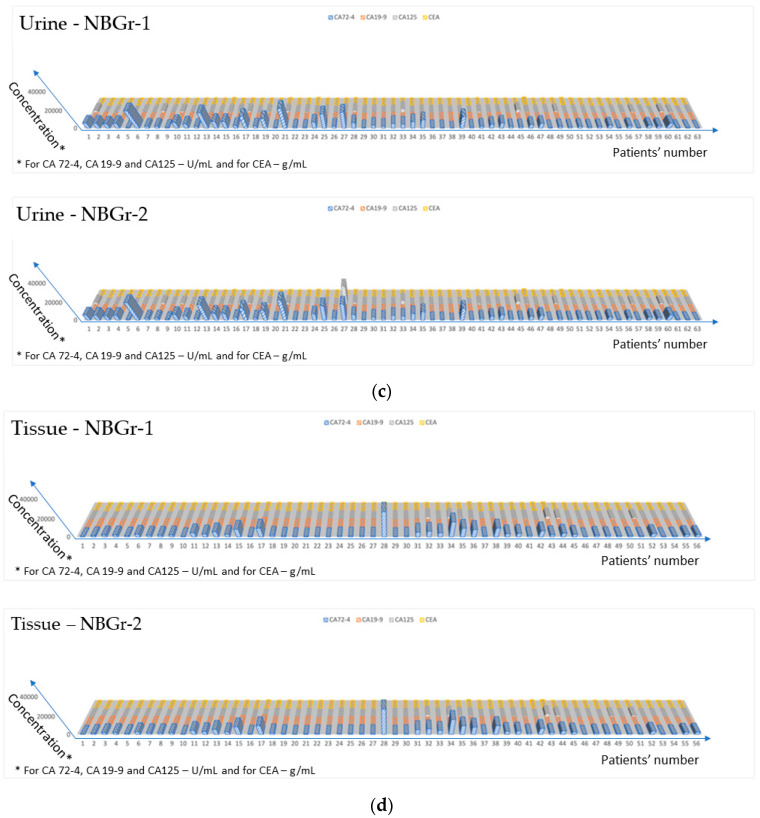
Comparison between the results obtained using the the two-needle 3D stochastic microsensors based on NBGr-1 and NBGr-2 for the assay of the four biomarkers in (**a**) saliva, (**b**) whole blood, (**c**) urine and (**d**) gastric tumor tissue samples.

The samples were analyzed as soon as possible after they were taken from the patients. The diagrams were recorded, and the first step was to identify, based on their signatures, the biomarkers CA 72-4, CA 19-9, CEA and CA 125 in the diagram. In between two signatures, the t_on_ was read. The values of t_on_ were used for the quantitative determination of CA 72-4, CA 19-9, CEA and CA 125 in whole blood, saliva, urine and tumoral tissue, according to the stochastic mode procedure described above. The levels of the four biomarkers were evaluated in all four types of biological fluids (whole blood, saliva, urine and tissue samples) with both sensors, and the results are given in Figure 4.

Student’s paired *t*-test was performed at a 99.00% confidence level (tabulated theoretical t-value: 4.032) for each biomarker. All calculated t-values were less than 3.500, which is less than the tabulated value, proving that there is no statistically significant difference between the results obtained using the two-needle 3D stochastic microsensors. The F-test was also performed, at a 95% confidence level, for ten samples of each kind. The tabulated F value was 3.18. The results obtained when comparing the standard deviations obtained for the two-needle 3D stochastic microsensors were lower than 1.00, which is a lower value than the tabulated value, 3.18. This indicates that there is no significant difference in the precision recorded for the two-needle 3D stochastic sensors, and that the standard deviations do not depend on the analyzed samples. Accordingly, the proposed microsensors can be used for the screening of whole blood, saliva, urine and tumoral tissues for the four biomarkers.

The second test performed for the validation of the needle 3D stochastic sensors and screening method was the recovery test. Known amounts of each of the biomarkers (CA 72-4, CA 19-9, CEA and CA 125) were added to whole blood, urine, saliva and tumoral tissues. The amounts of CA 72-4, CA 19-9, CEA and CA 125 were determined before and after their addition into the whole blood, urine, saliva and tumoral tissue samples. The difference between the final amount found into the biological sample and the initial amount (determined before the addition of known amounts of CA 72-4, CA 19-9, CEA and CA 125 to the sample) was compared with the known amount added into the sample for each of the biomarkers. The results obtained for the recovery tests are shown in Table 2.

The results presented in Table 2 show high recovery values for all biomarkers (CA 72-4, CA 19-9, CA 125 and CEA) when recovered from whole blood, saliva, urine and tumoral tissue samples. Very low % RSD values were also reported. 

Based on the first and second validation tests, one can conclude that the proposed needle 3D stochastic microsensors can be reliably used for the simultaneous assay of CA 72-4, CA 19-9, CA 125 and CEA in whole blood, saliva, urine and tumoral tissue. The test may be used as mass screening test of population for the early diagnosis of gastric cancer. 

## 4. Conclusions

The proposed needle 3D stochastic microsensors were successfully used for the assay of the four biomarkers CA 72-4, CA 19-9, CEA and CA 125 in urine, saliva, whole blood and gastric tumor tissue. The proposed stochastic sensor has been shown to be reliable for pattern recognition of the four biomarkers in biological samples from patients at very early stages, as well as patients at later stages, of gastric cancer. The reliable identification of CA 72-4, CA 19-9, CEA and CA 125 in urine, saliva, whole blood and gastric tumor tissue samples, followed by a highly reliable quantitative assay of CA 72-4, CA 19-9, CEA and CA 125 in urine, saliva, whole blood and gastric tumor tissue samples, suggest that the proposed needle 3D stochastic sensors are very good candidates for the mass screening of populations, especially as they are cost-effective and can be used for more than 100 measurements without renewing the microsensors’ active surfaces. Therefore, the main feature of these needle 3D stochastic microsensors is their utilization as tools in screening tests for the early detection of gastric cancers as well as in surgery rooms for the fast assessment of tissues, in order to decide on-site the way surgery and further treatment must proceed, in order to save the lives of patients.

## Figures and Tables

**Table 1:** Response characteristics of the stochastic microsensors used for the simultaneous assay of CA 72-4, CA 19-9, CA 125 and CEA.

Needle 3D Stochastic Microsensor Based on	Biomarker	Signature t_off_ (s)	Equation of Calibration, r	Sensitivity	Limit of Quantification	Linear Concentration Range
NBGr-2	CA 72-4 ^1^	1.6	1/t_on_ = 0.04 + 1.06 × 10^−5^ C r = 0.9923	1.06 × 10^−5^ s^−1^ U^−1^ mL	4.00 × 10^−11^ s^−1^ U^−1^ mL	4.00 × 10^−11^–4.00 × 10^3^ s^−1^ U^−1^ mL
	CA 19-9 ^1^	1.8	1/t_on_ = 0.03 + 1.82 × 10^−4^ C r = 0.9973	1.82 × 10^−4^ s^−1^ U^−1^ mL	3.28 × 10^−9^ s^−1^ U^−1^ mL	3.28 × 10^−9^–5.00 × 10^2^ s^−1^ U^−1^ mL
	CA 125 ^1^	1.4	1/t_on_ = 0.03 + 9.92 × 10^−5^ C r = 0.9994	9.92 × 10^−5^ s^−1^ U^−1^ mL	8.36 × 10^−6^ s^−1^ U^−1^ mL	8.36 × 10^−6^–837.43 s^−1^ U^−1^ mL
	CEA ^2^	1.1	1/t_on_ = 0.03 + 8.13 × 10^5^ C r = 0.9861	8.13 × 10^5^ s^−1^ g^−1^ mL	4.10 × 10^−15^ s^−1^ g^−1^ mL	4.10 × 10^−15^–2.00 × 10^−7^ s^−1^ g^−1^ mL
NBGr-1	CA 72-4 ^1^	1.0	1/t_on_ = 0.04 + 1.43 × 10^−5^ C r = 0.9977	1.43 × 10^−5^ s^−1^ U^−1^ mL	4.00 × 10^−3^ s^−1^ U^−1^ mL	4.00 × 10^−3^–4.00 × 10^3^ s^−1^ U^−1^ mL
	CA 19-9 ^1^	1.4	1/t_on_ = 0.04 + 5.88 × 10^−3^ C r = 0.9997	5.88 × 10^−3^ s^−1^ U^−1^ mL	2.09 × 10^−13^ s^−1^ U^−1^ mL	2.09 × 10^−13^–20.00 s^−1^ U^−1^ mL
	CA 125 ^1^	1.2	1/t_on_ = 0.04 + 1.98 × 10^−5^ C r = 0.9955	1.98 × 10^−5^ s^−1^ U^−1^ mL	8.37 × 10^−14^ s^−1^ U^−1^ mL	8.37 × 10^−14^–8.37 × 10^3^ s^−1^ U^−1^ mL
	CEA ^2^	1.6	1/t_on_ = 0.06 + 9.24 × 10^4^ C r = 0.9946	9.24 × 10^4^ s^−1^ g^−1^ mL	1.28 × 10^−11^ s^−1^ g^−1^ mL	1.28 × 10^−11^–1.00 × 10^−6^ s^−1^ g^−1^ mL

^1^ <C> = U mL^−1^ <t_on_> = s; ^2^ <C> = g mL^−1^ <t_on_> = s.

**Table 2:** Recovery tests of CA72-4, CA19-9, CA125 and CEA performed using the needle 3D stochastic microsensors (N = 10).

Needle 3D Stochastic Microsensor Based on	Biomarker	Whole Blood	Saliva	Urine	Tumoral Tissue
NBGr-2	**%, Recovery**				
	CA72-4	98.99 ± 0.03	99.43 ± 0.04	99.29 ± 0.03	97.25 ± 0.05
	CA19-9	97.23 ± 0.05	98.21 ± 0.03	98.14 ± 0.03	98.01 ± 0.03
	CA125	98.47 ± 0.02	99.12 ± 0.05	98.37 ± 0.04	97.15 ± 0.04
	CEA	99.05 ± 0.02	99.54 ± 0.05	98.11 ± 0.02	98.00 ± 0.04
NBGr-1	CA72-4	99.09 ± 0.08	99.01 ± 0.01	99.12 ± 0.02	98.29 ± 0.08
	CA19-9	99.00 ± 0.02	97.59 ± 0.04	99.87 ± 0.07	97.97 ± 0.09
	CA125	98.32 ± 0.05	98.06 ± 0.02	98.93 ± 0.04	98.19 ± 0.04
	CEA	98.27 ± 0.03	98.89 ± 0.05	98.90 ± 0.03	98.05 ± 0.02

## Data Availability

Not applicable.
